# Investigating the roles of medial prefrontal and superior temporal cortex in source monitoring

**DOI:** 10.1016/j.neuropsychologia.2018.10.001

**Published:** 2018-11

**Authors:** Peter Moseley, Kaja J. Mitrenga, Amanda Ellison, Charles Fernyhough

**Affiliations:** a Psychology Department, Durham University, Durham, UK; b School of Psychology, University of Central Lancashire, Preston, UK

**Keywords:** Neurostimulation, Source memory, Reality monitoring, tDCS, Hallucinations

## Abstract

Source monitoring, or the ability to recall the origin of information, is a crucial aspect of remembering past experience. One facet of this, reality monitoring, refers to the ability to distinguish between internally generated and externally generated information, biases in which have previously been associated with auditory verbal hallucinations in schizophrenia. Neuroimaging evidence suggests that medial prefrontal and superior temporal (STG) regions may play a role in reality monitoring for auditory verbal information, with evidence from a previous neurostimulation experiment also suggesting that modulation of excitability in STG may affect reality monitoring task performance. Here, two experiments are reported that used transcranial direct current stimulation (tDCS) to modulate excitability in medial prefrontal and superior temporal cortex, to further investigate the role of these brain regions in reality monitoring. In the first experiment (N = 36), tDCS was applied during the encoding stage of the task, while in the second experiment, in a separate sample (N = 36), it was applied during the test stage. There was no effect of tDCS compared to a sham condition in either experiment, with Bayesian analysis providing evidence for the null hypothesis in both cases. This suggests that tDCS applied to superior temporal or medial prefrontal regions may not affect reality monitoring performance, and has implications for theoretical models that link reality monitoring to the therapeutic effect of tDCS on auditory verbal hallucinations.

## Highlights

• Prior research suggests that tDCS applied to the left STG/TPJ affects source monitoring.
• We conducted 2 tDCS studies, stimulating during encoding or test of a source memory task.
• There was no effect on task performance when tDCS was applied to PFC or STG.

## 1 Introduction

The ability to recall the origin, or ‘source’, of information is a crucial aspect of remembering past experiences, and has been termed ‘source monitoring’ (Johnson et al., 1993). Source monitoring can be separated into various categories, depending on the sources of information that must be distinguished. For example, internal source monitoring requires the participant to distinguish between two or more internal sources (e.g., imagining a word, or speaking a word aloud), while external source monitoring requires the participant to distinguish between two or more sources external to the self (e.g., whether a word was spoken by one person or another person). The ability to recall whether information was externally generated (i.e., emanated from the surrounding environment) or internally generated (an action performed or imagined by oneself) is usually referred to as ‘reality monitoring’ (Johnson et al., 1993, Johnson and Raye, 1981, Mitchell and Johnson, 2009).

Typically, reality monitoring is assessed using a source memory paradigm, in which the participant must recall whether a stimulus (e.g., a word or image) was previously presented to them, or whether they imagined/spoke the stimulus themselves (Mitchell and Johnson, 2009). Research falling under the source monitoring framework has thus investigated the specific qualitative attributes of memories that may contribute to judgements of source; for example, an event represented in memory as especially vivid (i.e., high in perceptual detail) may be more likely to be recalled as originating from the external environment (Johnson et al., 1988, Sugimori et al., 2014). Conversely, the source of a remembered event associated with higher cognitive load may be more easily recalled (Krefeld-Schwalb et al., 2015), and more likely to be recalled as self-generated, or ‘internal’ (Finke et al., 1988).

Failures in processes associated with reality monitoring are thought to be associated with a number of psychiatric and neurological conditions (e.g., Barnes et al., 2003; Olson et al., 2016), notably including hallucinatory experiences in which an individual mistakes an internally generated mental image (e.g., inner speech; Alderson-Day and Fernyhough, 2015; Moseley et al., 2013) for an external percept (Ditman and Kuperberg, 2005, Jones and Fernyhough, 2007, Seal et al., 2004). For example, a number of studies have shown biased performance on reality monitoring tasks in hallucinating individuals with a diagnosis of schizophrenia, compared to patients with no hallucinations, and healthy controls (e.g., Bentall et al., 1991; Woodward et al., 2007; see Brookwell et al., 2013, for a meta-analysis). One possibility highlighted by the source monitoring framework is that individuals who hallucinate generate excessively vivid mental imagery (Aleman et al., 2003), which is therefore more likely to become misattributed. This is consistent with neuroimaging evidence for superior temporal gyrus (STG) activation in both vivid verbal imagery and during AVH (Jardri et al., 2011, Zvyagintsev et al., 2013). An alternative, though not exclusive, possibility, is that atypical efference copy mechanisms relating to sensory predictions of self-generated motor acts cause internally generated events to be misattributed to an external source (Ford and Mathalon, 2005, Frith, 1992), a possibility that has received empirical support from neuroimaging studies showing that, when vocalising, patients with a diagnosis of schizophrenia show reduced cortical attenuation in the STG compared to controls – a pattern not evident when participants simply listened to the same vocalisations played back (Ford et al., 2001, Ford et al., 2007).

Neuroimaging evidence has also suggested a role for the STG in source memory tasks using auditory verbal information. In a paradigm that required participants to recall whether words had previously been heard or only imagined, Sugimori et al. (2014) showed that frontal regions including the middle frontal gyrus were more active during encoding of words that were correctly recalled as ‘imagined’. This could be interpreted as evidence that engagement of cognitive operations (reflected in MFG activity) later acted as a cue that stimuli were self-generated. Sugimori et al. also investigated the link between reality monitoring, cortical activation, and the tendency of the participants to experience auditory hallucinations (assessed using a self-report measure), finding that activity in the STG (encompassing primary and secondary auditory cortex) when participants externally misattributed a word they had imagined as ‘heard’ was significantly correlated with the tendency to experience auditory hallucinations. This is consistent with neuroimaging evidence suggesting that primary and secondary auditory cortical regions are active during the experience of auditory verbal hallucinations (AVH) (Jardri et al., 2011).

Cortical regions such as medial prefrontal cortex also seem to play a crucial role in both encoding and recognising the source of information, with evidence suggesting that anterior medial prefrontal cortex (amPFC) plays a specific role in monitoring internally- versus externally-generated information, as is required in reality monitoring (Mitchell and Johnson, 2009, Vinogradov et al., 2006). For example, Simons et al. (2006) showed that, during the retrieval (test) stage of a source memory paradigm, reduced activation in the amPFC was associated with the likelihood that imagined events would be incorrectly recollected as perceived. Furthermore, structural imaging has shown that absence of the paracingulate sulcus (located adjacent to, and associated with differential grey matter volumes in, the medial PFC) is associated with impaired reality monitoring performance (Buda et al., 2011) and length of the paracingulate sulcus with the presence of hallucinations in schizophrenia (Garrison et al., 2015). It has been theorised that activity in PFC regions may reflect engagement of higher level cognitive operations and so may be important in feelings of effort associated with self-generated events (Sugimori et al., 2014). For example, drawing on evidence relating anterior medial PFC activity to, among other things, metacognition, theory of mind, and the default mode network, the ‘gateway hypothesis’ specifies that anterior PFC plays a role in switching between stimulus-oriented (i.e., external) and stimulus-independent (i.e., internal) thought (Burgess et al., 2005). It is therefore no surprise that amPFC has been implicated in the ability to retrospectively distinguish between events that were external and events that were internal.

Furthermore, there is evidence that transcranial direct current stimulation (tDCS), a noninvasive brain stimulation technique, can reduce the frequency of AVH in hallucinating schizophrenia patients (Brunelin et al., 2012, Mondino et al., 2015; though see Fitzgerald et al., 2014, for a null result), attenuate cortical activity in response to self-produced vocalisations (Nawani et al., 2014), and modulate false perceptions in a non-clinical population (Moseley et al., 2014), when applied in a frontotemporal montage (i.e., with electrodes positioned over prefrontal and superior temporal regions). A recent tDCS study attempted to link findings regarding the neural basis of reality monitoring and auditory hallucinations, by modulating reality monitoring performance using tDCS applied to the left STG, in a non-clinical population. Mondino et al. (2016) placed the cathodal electrode (aiming to decrease cortical excitability) over the PFC in one group of participants, and the anodal electrode (aiming to increase cortical excitability) over the left TPJ in a second group, stimulating throughout both encoding and test phases of the task. In both cases, the reference electrode was positioned over occipital cortex. Compared to an active control condition and sham condition, they found that anodal stimulation to the left TPJ selectively disrupted reality monitoring (but not internal source monitoring), causing participants to incorrectly recall more imagined items as heard. No effect on reality monitoring performance was found following stimulation to the PFC. This study therefore suggested that stimulation of posterior superior temporal regions can affect reality monitoring for auditory verbal information, presumably due to modulation of excitability in this region, or due to effects on other regions distal to the stimulating electrodes. Given that the researchers stimulated throughout the entirety of the reality monitoring task, however, it is not possible to tell whether the effect of stimulation was due to an effect on encoding or retrieval during the source memory task. The effect of stimulation could feasibly result from modulating encoding (e.g., directly affecting perceptual vividness via stimulation of the STG, or by interfering with processes involved in the sense of agency, via stimulation of the immediately adjacent temporoparietal junction) or retrieval (e.g., interfering with recall of perceptual information).

Nevertheless, Mondino et al.’s study is important because it potentially provides a link between the therapeutic effect of neurostimulation (i.e., reducing the frequency of AVH), and the underlying cognitive mechanisms (i.e., biased reality monitoring). Furthermore, one study showed a concurrent improvement in performance on a source memory task alongside a reduction in AVH frequency following treatment using offline 1 Hz rTMS (Brunelin et al., 2006), albeit with a small sample size (*N* = 24 in a between groups design). Given the link between AVH and reality monitoring, this may provide a mechanism by which tDCS could have a therapeutic effect on AVH, as has been previously speculated (Moseley et al., 2013). A recent study also showed that transcranial random noise stimulation (tRNS, a variant of tDCS), applied to the mPFC and occipital cortex, modulated reality monitoring performance, although in contrast to Mondino et al.’s study, the effect was only observed in older adults, and not a group of younger adults (Mammarella et al., 2017). In contrast to previous research, this study only stimulated during the learning stage of the task, implying that the effect of stimulation is through modulation of encoding.

The present study therefore further tested the involvement of two cortical regions in reality monitoring for auditory verbal stimuli: the right anterior medial prefrontal cortex and the left superior temporal gyrus. We used tDCS to increase or decrease cortical excitability whilst participants completed the encoding stage (Experiment 1) or the test stage (Experiment 2) of a source memory task. The rationale for applying stimulation during the encoding stage was based on models of reality monitoring suggesting that vividness of self-generated imagery, and cognitive operations associated with the imagery generation, may underlie later source judgements (see the aforementioned study by Sugimori et al., 2014). Alternatively, stimulation of STG or amPFC may affect retrieval of relevant source information, with neuroimaging evidence from Simons et al. (2006), for example, suggesting that amPFC activity during retrieval is associated with accuracy of source judgements. The study therefore aimed to extend upon Mondino et al. (2016)’s study, that stimulated throughout both task stages (and therefore could not provide evidence for an effect of stimulation on a specific part of the task). A frontotemporal montage was contrasted to a frontal-occipital montage and a sham condition. In both experiments, tDCS was applied in three conditions: 1) with the anodal electrode positioned over the left STG and the cathodal electrode positioned over the right amPFC (frontotemporal condition); 2) with the anodal electrode positioned over the right occipital lobe (as a control site) and the cathodal electrode positioned over the right amPFC (frontal-occipital condition); 3) positioned as in the first condition, but only applied for a short period (sham condition). For Experiment 1, it was hypothesized that frontotemporal stimulation would lead to a misattribution bias in source judgements, such that participants were more likely to incorrectly recall that imagined words had been heard, compared to other stimulation conditions (as found by Brunelin et al.). Given the role of amPFC in reality monitoring (albeit usually implicated in retrieval), however, the frontal-occipital condition was included as an active control condition, to test whether any effects could be solely due to stimulation of the amPFC. For Experiment 2 (stimulation during test), since more evidence points towards involvement of the amPFC in retrieval of source judgements, it was hypothesized that both active stimulation conditions would lead to an overall reduced reality monitoring accuracy (as opposed to a bias) in making source judgements, compared to sham stimulation. However, if frontotemporal stimulation during test reduced reality monitoring accuracy compared to all other conditions, this would imply a specific role for the STG in source retrieval for auditory verbal information. Additionally, we conducted further analysis of data from both experiments together, regarding task performance, imagery vividness, and self-reported hallucination-proneness.

## 2 Experiment 1

### 2.1 Materials and methods

#### 2.1.1 Participants

The sample consisted of 36 participants (9 males, 27 females), aged 18–28 (*M* = 20.14, *SD* = 2.5). All participants were right-handed, and were considered ineligible to take part if they reported any hearing problems or any history of neurological or psychiatric disorder. Participants were also screened for contra-indications for neurostimulation (e.g., history of epilepsy, recurring skin conditions, non-removable metallic objects on the head). Ethical approval was provided by Durham University Ethics Committee, and written informed consent was given by participants, in accordance with the Declaration of Helsinki. All participants were rewarded with a £25 gift voucher for participating, as well as course credits if required. The study was advertised as testing ‘auditory memory’.

#### 2.1.2 Source memory task

The source memory task was based upon that used by Sugimori et al. (2014). The task consisted of two stages: the encoding stage and the test stage. The stimuli consisted of 450 words taken from the MRC Psycholinguistic Database (Wilson, 1988), each consisting of 1–3 syllables and 4–6 letters. The words were separated into nine lists, each consisting of 50 words, matched on mean number of letters and syllables, as well as Kucera-Francis frequency, familiarity, concreteness, imageability, and meaningfulness.

In the encoding stage, participants were presented with a series of 100 words, taken from two lists. One list was assigned to be ‘heard’ and the other list ‘imagined’. Participants were presented with each word consecutively. The heard and imagined words were presented alternately (i.e., Hear-Imagine-Hear-Imagine) to ensure that different encoding conditions were balanced across time during stimulation, although the order in which the items from each list were presented was randomised for each participant. Immediately before each word, participants were cued with the word ‘HEAR’ or ‘IMAGINE’ in the centre of the screen. Each word was then presented for 2500 ms in the centre of the screen. If the word followed the cue to ‘hear’ the stimulus, it was accompanied by an auditory stimulus, of a male voice speaking the word once. If the participant had been cued to imagine the presented word, no auditory stimulus was presented, and the participant was required to imagine hearing the word being read out in the same male voice they had heard. Following the presentation of each word, the participant was cued to provide a rating for how vividly they had heard/imagined the word (on a scale from 1 to 3, as used by Sugimori et al.). Specifically, participants were informed that they should rate each word for how ‘clear, detailed and realistic’ it had sounded. The rating screen was presented for 2.5 s, regardless of whether a response was entered. The encoding stage therefore lasted a total of 900 s.

Following the encoding stage, participants were given a short break from the task (in which time the tDCS electrodes were removed from their scalp). Participants were either given a break of 5 min or 15 min. We included this between-subject variable because neurostimulation techniques such as tDCS can have after-effects beyond the period of stimulation, both at a neural (Stagg and Nitsche, 2011) and behavioural (Hummel and Cohen, 2006) level, which may have directly affected task performance in the test stage (as opposed to indirectly through the effect of stimulation on encoding that we wished to study). If any observed effect was due to after-effects of the tDCS, we would expect it to be weaker after a longer time period, as the effects of stimulation began to wear off. During the task break, participants were asked to sit quietly in the darkened room; it was not appropriate to give participants a distractor task, since this may have interacted with the effects of the tDCS.

After the task break, participants completed the test stage of the task. In this stage, they were presented with the two lists of words included in the encoding stage, as well as a third list which had not been previously presented. In this stage, they were therefore presented with the 50 words which had previously been heard, the 50 words which had previously been imagined, and 50 words which had not previously been presented. The words were presented in a random order, and appeared on the screen until a response was entered. For each word, participants were asked to respond, with a button press, whether they believed the word had been heard or imagined in the first stage, or if the word was completely new.

#### 2.1.3 Other measures

Participants completed a revised version of the Launay-Slade Hallucination Scale (LSHS; as used in McCarthy-Jones and Fernyhough, 2011), a self-report measure of hallucination-proneness that asks participants to rate the frequency of hallucinatory experiences (e.g. ‘I have had the experience of hearing a person's voice and then found that no-one was there.’). The scale consists of 9 items, with each item scored between 1 and 4, summing to a possible total of 36.

Finally, at the end of the third session, participants completed a short questionnaire aimed at assessing the efficacy of the sham stimulation condition. After being informed that one of the three sessions did not consist of stimulation for the full 15 min, participants were asked which session they thought this might be (and were prompted to guess if they did not know), and then asked to rate their confidence about this decision on a scale from 1 to 7 (1 = not confident at all; 4 = somewhat confident; 7 = very confident).

#### 2.1.4 Transcranial direct current stimulation

Participants received 900 s (plus 8 s of fade-in and 8 s of fade-out) of tDCS whilst completing the encoding stage of the source memory task, using a Magstim Eldith DC Stimulator. A 1 mA current was delivered through two 5 × 5 cm (25 cm^2^) electrodes, placed in sponges soaked in 0.9% NaCl solution, and held in place on the participant's scalp by two rubber straps. There were three separate stimulation conditions, which all participants completed. In these, three regions were stimulated: right anterior medial PFC (amPFC), left STG, and visual area V5/MT (the latter was chosen as a control site, because it was not expected to play a role in the source memory task used in the present study). These sites were localised using the EEG 10–10 system, which adjusts for individual head size, and has previously been used to target the left STG under electrode site CP5 (e.g., Moseley et al., 2014; You et al., 2011) and right amPFC under electrode FP2 (e.g., Karim et al., 2010). The V5 electrode was positioned 3 cm above the inion, and 6 cm right of the midline, as in previous studies which have stimulated this cortical region (Antal et al., 2004, Ellison et al., 2007).

Participants therefore received stimulation on three occasions. In one session, the cathodal electrode was positioned over the right amPFC, and the anodal electrode over the left STG (subsequently referred to as the ‘frontotemporal’ condition). In another session, the cathodal electrode was positioned over the right amPFC, and the anodal electrode over left V5 (subsequently referred to as the ‘frontal-occipital’ condition). Finally, in another session, the electrodes were positioned as in the frontotemporal condition, but stimulation was only applied for 30 s, plus 8 s fade-in and 8 s fade-out (subsequently referred to as the ‘sham’ condition). Sham stimulation is not sufficient to modulate neuronal excitability, and has previously been demonstrated to be an effective method of blinding participants to the condition (Gandiga et al., 2006). This method of sham stimulation is possible because tDCS emits no sound and evokes only a mild tingling sensation underlying the electrodes. The study was conducted as a single-blind experiment (that is, the experimenter was aware of the stimulation condition, but the participant was not). Although a double-blind procedure is preferable, this approach would be difficult within the present methodology, due to the different electrode montages used across different conditions. Where possible, sessions were separated by 7 days (mean no. days between Sessions 1–2 = 7.17, *SD* = 0.51, range: 7–9; mean no. days between Sessions 2–3 = 6.92, *SD* = 0.55, range: 5–8). The order in which participants completed each condition was counterbalanced.

#### 2.1.5 Procedure

In each session, participants were seated in front of a computer and provided with earbuds (Creative EP-630), through which the stimuli were played. After the task was described, the tDCS electrodes were placed on the participants scalp, although before the stimulation commenced, participants listened to a brief sound clip, consisting of the male voice used in the source memory task reading a short extract from a book (60 s). This was so that the participants had enough prior experience of hearing the voice stimulus to be able to imagine the words included in the task in that voice. The passage was edited so that it did not include any of the words from the source memory task. Participants then completed a short practice task of both stages of the task (consisting of 6 words in the encoding stage, then 9 words in the test stage). Participants were allowed to repeat the practice stage if they wished. The tDCS was then started, and after the 8 s fade-in period, the encoding stage of the task began. After 900 s, the encoding stage of the task ended, as the fade-out period (8 s) of the tDCS began. The participant was then asked to sit quietly for either 5 or 15 min (see above), in which time the electrodes were removed from their scalp. They then completed the test stage of the task, followed by the hallucination-proneness measure (LSHS) and sham efficacy measure (final session only).

This procedure was kept identical across all three sessions (i.e., participants listened to the example sound clip and completed the practice task each time they attended). The only difference between each session was that 1) different word lists were used in each session; 2) the stimulation condition was varied.

#### 2.1.6 Data analysis

Power analysis (conducted using G*Power 3.1) indicated that, for the main effect of stimulation condition (within subjects), to obtain 80% power with an effect size of η^2^ = 0.15 (as obtained by Mondino et al., 2016) and an alpha level of 0.05, a sample size of 30 was needed. For counterbalancing, 36 participants were recruited into the present experiment, with all participants completing all three stimulation conditions in separate sessions. All further data analysis was conducted using JASP.

Data analysis mainly focused on two dependent variables: reality monitoring accuracy, and misattribution bias. Reality monitoring accuracy was calculated as in previous papers using similar source memory paradigms (e.g., Garrison et al., 2017) as the total number of items correctly classified as heard or imagined divided by the number of items correctly classified as ‘old’. The proportion of externalisation errors (i.e., the proportion of errors on which an imagined item was classified as heard) was calculated as the total number of imagined items classified as heard, divided by the total number of imagined items classified as heard or new. The proportion of internalisation errors (i.e., judging a heard item as imagined) was calculated as the number of heard items classified as imagined, divided by the number of heard items classified as imagined or new. Misattribution bias was then calculated as externalisation errors minus internalisation errors, with positive values indicating a bias towards externalisation errors, and negative values indicating a bias towards internalisation errors. Additional analysis was conducted on old–new accuracy (to test whether any difference in task performance across conditions was specific to reality monitoring), calculated as the proportion of items correctly classified as previously presented (heard/imagined) or new.

Stimulation condition (frontotemporal/frontal-occipital/sham) was included as a within-subjects variable, whilst interval length (5 mins/15 mins) was a between-subjects variable. 3 × 2 analyses of variance were therefore carried out for reality monitoring accuracy, misattribution bias, and old–new accuracy (to check for an effect on general memory performance), to analyse the effect of tDCS on task performance. Greenhouse-Geisser corrections were applied in the event that assumptions of sphericity were broken (Mauchley's test, *p* < .05).

Further analysis was conducted using Bayesian analysis of variance, to assess whether there was evidence for the null hypothesis. This was conducted using JASP, with default priors in all cases. JASP default priors use Cauchy distributions (fixed effects *r* = 0.5) peaking at 0 (i.e., 50% of the prior distribution falls between -0.5 and 0.5). Cauchy distributions are similar to normal distributions, but have fatter tails and have been recommended for use with Bayes factors. Bayes factors are then calculated, representing the ‘predictive adequacy’ of the posterior over the prior model (see Wagenmakers et al., 2017, for further information). Conclusions regarding evidential strength were based on the classifications proposed by Wagenmakers et al. (2017), with BF_10_ > 1 and < 3 interpreted only as ‘anecdotal’ evidence for the alternative hypothesis, BF_10_ < 1 and > 0.33 interpreted only as ‘anecdotal’ evidence for the null hypothesis, and BF_10_ of < 0.33 or > 3 interpreted as moderate, strong, or very strong, evidence.

### 2.2 Results

**Fig. 1:**
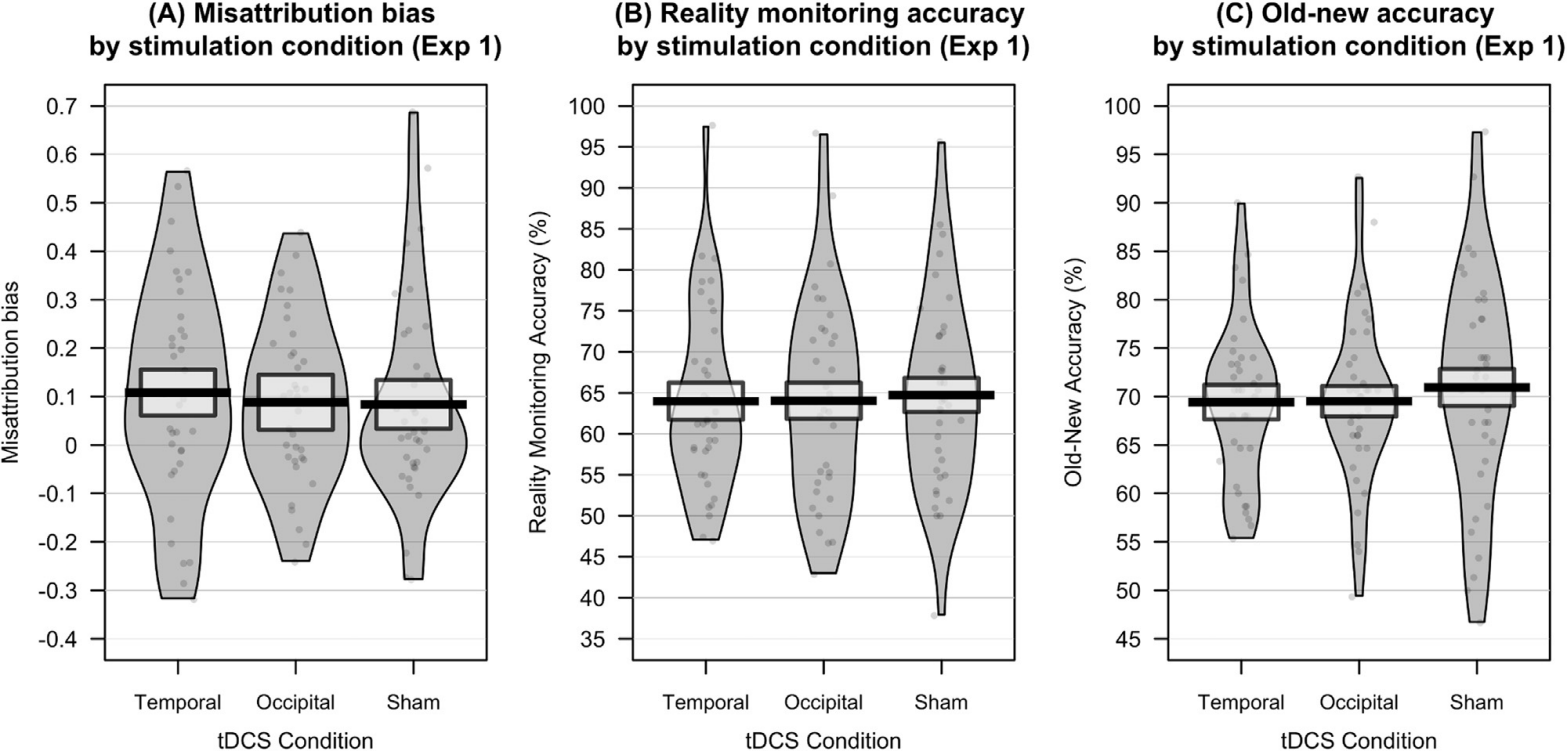
A: Misattribution bias: higher values correspond to a higher likelihood of external misattributions when errors are made. B: Reality monitoring accuracy: the percentage of trials on which participants made correct source judgements, for items correctly recalled as old. C: Old–new accuracy: the percentage of trials on which participants correctly classified items as old or new. Solid line = mean performance. Box = 95% confidence interval. Violin = smoothed density. Violin plots were generated in R, using the ‘yarrr’ package.

Descriptive statistics and data visualisation for task performance, by stimulation condition, are presented in Fig. 1 and Table 1. Missed responses for vividness ratings was low (< 3% in all conditions).

**Table 1:** Performance on source memory task, by tDCS condition, in Experiment 1 (M, SD).

	Temporal	Occipital	Sham
Reality monitoring accuracy (%)	63.98 (11.32)	64.04 (12.30)	64.74 (12.00)
Misattribution bias	0.108 (0.23)	0.088 (0.17)	0.084 (0.21)
Old-new accuracy (%)	69.43 (8.28)	69.52 (8.88)	70.94 (11.73)

There was no main effect of stimulation condition on misattribution bias (*F*(2, 68) = 0.27, *p* = .768, η^2^ = .008), although there was a main effect of interval length (*F*(1, 34) = 5.50, *p* = .025, η^2^ = .139), with misattribution bias after a 5 min interval length (*M* = 0.15, *SD* = 0.16) significantly higher than after a 15 min interval length (*M* = 0.03, *SD* = 0.16); that is, when participants made errors, they were more biased towards external misattributions after a 5 min interval, compared to a 15 min interval. There was no interaction between stimulation condition and interval length for misattribution bias (*F*(2, 68) = 0.74, *p* = .483, η^2^_p_ = .021). Bayesian analysis indicated that there was anecdotal evidence for the model in which there was only a main effect of interval length (BF_10_ = 2.51). Meanwhile, there was moderate evidence for the null hypothesis for the main effect of stimulation condition (BF_10_ = 0.11) and for a model in which there was no main effect of stimulation condition or interval length (BF_10_ = 0.28). Finally, there was strong evidence for a model in which there was no effect of either stimulation condition or interval length, and no interaction between the two (BF_10_ = 0.07). Bayesian analysis therefore provides strong evidence for the null hypothesis (i.e., evidence that there was no effect of tDCS on misattribution bias, nor any interaction with interval length).

There was also no main effect of stimulation condition on reality monitoring accuracy (*F*(2, 68) = 0.15, *p* = .861, η^2^ = 0.004), nor a main effect of interval length on reality monitoring accuracy (*F*(1, 34) = 1.08, *p* = .306, η^2^ = 0.031). There was no interaction between stimulation condition and interval length (*F*(2, 68) = 0.20, *p* = .817, η^2^_p_ = 0.006). Bayesian analysis provided very strong evidence for a model in which there were no main effects or interactions (BF_10_ = 0.01). There was also strong evidence for the model in which there was no main effect of stimulation (BF_10_ = 0.10) and the model in which there was no main effect of both stimulation condition and interval length (BF_10_ = 0.06), although only anecdotal evidence for the model in which there was no main effect of interval length (BF_10_ = 0.64). This analysis therefore provides evidence for the null hypothesis (i.e., no effect of tDCS on reality monitoring accuracy).

Finally, there was no main effect of stimulation condition on old–new accuracy (*F*(2, 68) = 0.96, *p* = .388, η^2^ = .027), nor a main effect of interval length (*F*(1, 34) = 0.64, *p* = .431, η^2^ = .018). There was no interaction between stimulation condition and interval length (*F*(2, 68) = 0.75, *p* = .477, η^2^_p_ = .021). Bayesian analysis provided very strong evidence for a model in which there were no main effects or interactions (BF_10_ = 0.03), and moderate evidence for a model in which there was no effect of stimulation condition or interval length (BF_10_ = 0.10) and a model in which there was no effect of stimulation condition (BF_10_ = 0.19). There was only anecdotal evidence for a model in which there was no main effect of interval length (BF_10_ = 0.56). Again, this analysis provides evidence that there was no effect of the experimental manipulation on old-new accuracy.

We also conducted further exploratory analysis to test for differences in the distributions of performance under different tDCS conditions, using a shift function (as recommended by Rousselet, Pernet, and Wilcox, 2017). The shift function compares scores at each quantile, as a function of the quantiles of one group. In this case, we inspected differences between deciles, using the sham stimulation condition as baseline. Therefore, confidence intervals were calculated (correcting for multiple comparisons) for differences between groups at each decile. Thus, the shift function can be used to assess whether a manipulation may affect specific parts of a distribution (e.g., participants who perform poorly on a task) (see Rousselet, Pernet, and Wilcox, 2017, for more information). We conducted this analysis separately with misattribution bias, reality monitoring accuracy, and old–new accuracy as dependent variables, and found little evidence of differences in distribution between stimulation conditions for any variables, with all 95% confidence intervals crossing 0 after corrections for multiple comparisons, with the exception of the comparison between sham and frontotemporal stimulation on old-new accuracy at the 8th decile (95% CI [0.53–10.33]; that is, old–new accuracy was lower in the frontotemporal stimulation condition in the 8th decile). Inspection of the shift function plots suggests that participants with higher scores in old–new accuracy showed comparatively reduced performance in both stimulation conditions compared to sham (see Supplementary materials for graphs illustrating shift functions), perhaps indicating that stimulation may have decreased overall recognition performance only in high performers. The present study is, however, perhaps underpowered to detect this difference after correction for multiple comparisons; the above analysis is presented only as an exploration of the data.

A 3 × 3 (actual sham session × guessed sham session) chi square analysis indicated that participants were able to guess the sham session at an above chance level (χ^2^(4) = 21.28, *p* < .001), with 24 out of 36 participants correctly choosing the sham session when asked at the end of the third session. The median confidence rating for choosing the sham session was 4 (corresponding to ‘somewhat confident’), although there was not a significant difference between the ratings of participants who guessed correctly (*Mdn* = 4), and those who did not (*Mdn* = 5) (W = 90.0, *p* = .066, *r*_rb_ = 0.38).

### 2.3 Interim summary

Experiment 1 tested the effect of tDCS applied to the left STG (anodal) and right amPFC (cathodal) during the encoding stage of a reality monitoring task, finding no difference in task performance between stimulation conditions. Further analysis using Bayes factors provided evidence for the null hypothesis (i.e., that there was no effect of stimulation on reality monitoring). In contrast to previous findings, these results suggest that modulating excitability of temporal or frontal regions during encoding does not affect subsequent source judgements. However, previous findings could potentially be explained by the effect of stimulation during source judgements (i.e., during the test stage of the task). For Experiment 2, stimulation was therefore applied during the test stage of the task, using otherwise similar tasks measures and stimulation parameters.

## 3 Experiment 2

### 3.1 Materials and methods

#### 3.1.1 Participants

The sample consisted of 36 participants (8 males, 28 females), aged 18–35 (*M* = 22.69, *SD* = 5.7), none of whom had taken part in Experiment 1. Participants met the same eligibility criteria as specified for Experiment 1, and were again rewarded with a £25 gift voucher and course credits for participation.

#### 3.1.2 Transcranial direct current stimulation

Stimulation was applied within the same parameters specified above (stimulation time = 900 s, fade-in/out = 8 s, electrode size = 25 cm^2^, current strength = 1 mA), although was used during the test stage of the task, as opposed to the encoding stage. Based on the data from Experiment 1, it was estimated that the test stage should take no more than 10 min to complete. Stimulation therefore commenced 5 min before task onset, during which time the participant was asked to sit quietly. Stimulation was switched off when the participant ended the test stage of the task.

#### 3.1.3 Procedure

Participants completed the same source memory task as in Experiment 1, with the sole difference being that the interval between the encoding and test stage was 5 min for all participants. This was because stimulation was applied only during the test stage, and therefore there was no possibility of stimulation affecting performance in subsequent stages of the task. The tDCS equipment was set up and electrodes placed upon the scalp prior to the start of the task, and was then turned on as soon as the encoding stage ended. Participants again participated in three sessions (frontotemporal, frontal-occipital, sham) separated by approximately one week in time (mean no. days between Sessions 1–2 = 6.92, *SD* = 0.84, range: 5–10; mean no. days between Sessions 2–3 = 7.17, *SD* = 1.14, range: 5–12). The order in which participants completed each condition was counterbalanced, as in Experiment 1.

#### 3.1.4 Data analysis

Data analysis proceeded as in Experiment 1, with the dependent variables of interest being reality monitoring accuracy and misattribution bias. Old–new accuracy was also compared across stimulation conditions. There was no between-subjects variable of interval length, since this was fixed to 5 mins across all participants.

### 3.2 Results

**Fig. 2:**
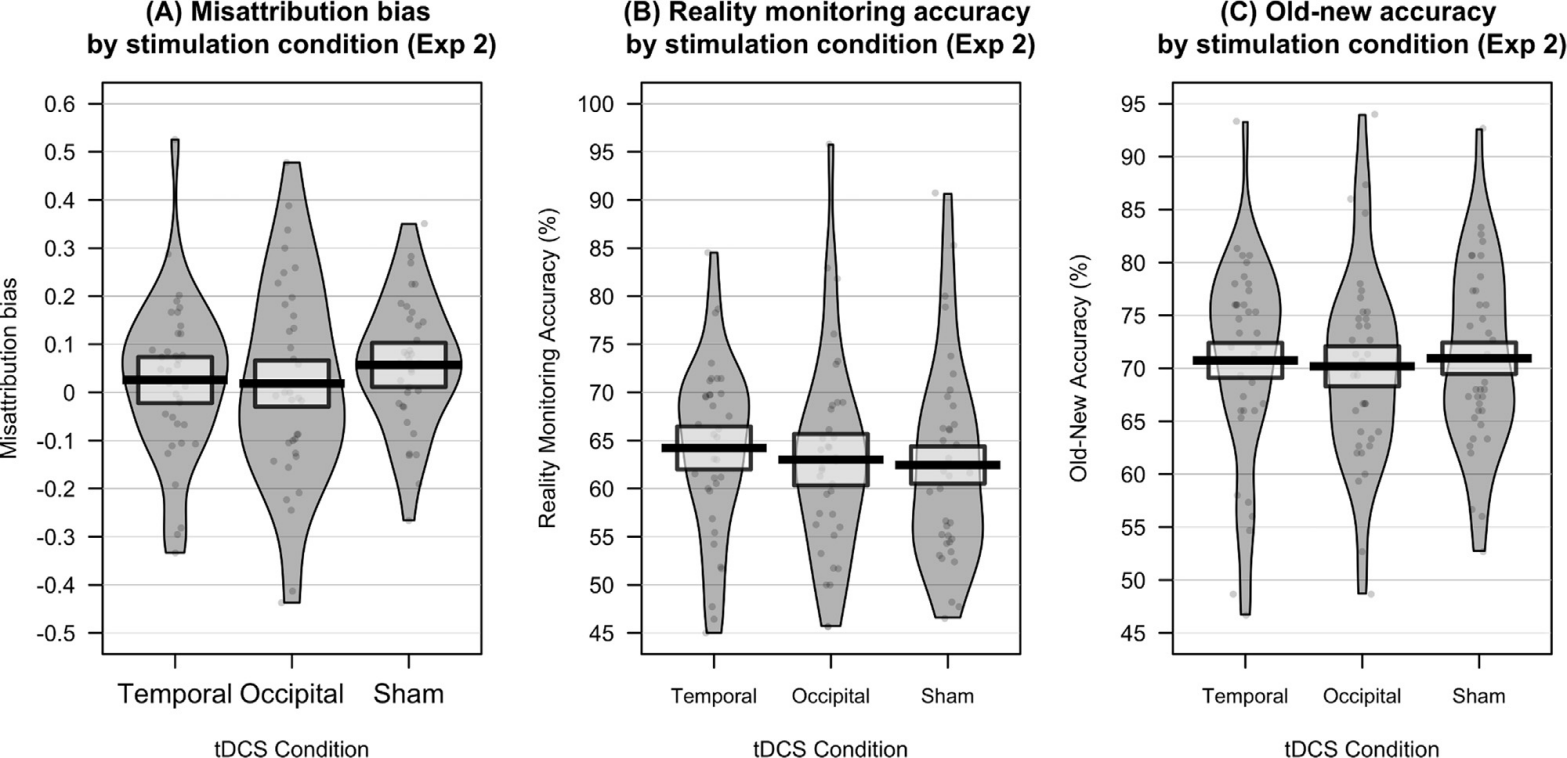
A: Misattribution bias: higher values correspond to a higher likelihood of external misattributions when errors are made. B: Reality monitoring accuracy: the percentage of trials on which participants made correct source judgements, for items correctly recalled as old. C: Old-new accuracy: the percentage of trials on which participants correctly classified items as old or new. Solid line = mean performance. Box = 95% confidence interval. Violin = smoothed density. Violin plots were generated in R, using the ‘yarrr’ package.

Descriptive statistics and data visualisation for task performance, by stimulation condition, are presented in Table 2 and Fig. 2.

**Table 2:** Performance on source memory task, by tDCS condition, in Experiment 2 (M, SD).

	Temporal	Occipital	Sham
Reality monitoring accuracy (%)	64.22 (9.06)	63.00 (10.56)	62.46 (10.42)
Misattribution bias	0.026 (0.17)	0.018 (0.21)	0.057 (0.14)
Old-new accuracy (%)	70.74 (9.74)	70.19 (9.31)	70.94 (8.66)

A repeated measures ANOVA, with stimulation condition (frontotemporal/frontal-occipital/sham) as a within-subjects variable, showed no main effect of stimulation on reality monitoring accuracy (*F*(2, 70) = 0.64, *p* = .531, η^2^ = .018) or misattribution bias (*F*(2, 70) = 0.79, *p* = .459, η^2^ = .022), nor on old–new accuracy (*F*(2, 70) = 0.23, *p* = .799, η^2^ = .006). In each case, Bayesian analysis provided moderate support for a model in which there was no main effect of stimulation on task performance (for reality monitoring accuracy, BF_10_ = 0.14; for misattribution bias, BF_01_ = 0.17; for old–new accuracy, BF_01_ = 0.10). This analysis therefore provides support for the null hypotheses (i.e., no effect of tDCS on any task measure).

As in Experiment 1, we conducted further exploratory analysis of misattribution bias, reality monitoring accuracy, and old–new accuracy, using shift functions (see Supplementary materials for shift function plots). Again, this analysis did not suggest any significant differences in distribution between performance in the different stimulation conditions, with all 95% confidence intervals between deciles crossing 0, with the exception of the comparison between performance in the sham and frontotemporal condition, in which confidence intervals in the 3rd decile did not cross 0 (95% CI [-9.45, -0.20]), suggesting a small improvement in reality monitoring accuracy in the frontotemporal condition at this decile. However, inspection of the shift function plot did not suggest any clear difference in the overall distribution of the two conditions (see Supplementary materials).

A 3 × 3 chi square analysis (actual sham session × guessed sham session) showed that participants did not guess the sham session at a level significantly above chance (χ^2^(4) = 9.13, *p* = .058), with 20 out of 36 guessing correctly, with a median confidence rating of 4 (‘somewhat confident’). As in Experiment 1, participants who guessed correctly (*Mdn* = 4.5) were not significantly more confident in their choice than those who guessed incorrectly (*Mdn* = 4) (W = 126.5, *p* = .283, *r*_rb_ = 0.21).

### 3.3 Interim summary

Experiment 2 used similar task measures and stimulation parameters as in Experiment 1, with the main difference being that tDCS was applied during the test stage of the task. Again, there was no difference in task performance between the three stimulation conditions, and Bayesian analysis provided evidence in support of the null hypothesis (no effect of stimulation), suggesting that stimulation of the STG and amPFC did not affect source judgements. Across two experiments, then, the present data provides no evidence of modulation of reality monitoring task performance, in contrast to previous studies (Mondino et al., 2016). Possible reasons for the different effects across studies include differences in experimental design such as task difficulty or stimulation parameters, which are discussed further in Section 5, below.

## 4 Further analysis: hallucination-proneness, vividness of imagery, and reality monitoring performance

The source monitoring framework suggests that high levels of vividness of auditory verbal imagery is one factor that leads to internally generated stimuli being misattributed to an external source (Johnson et al., 1993, Johnson et al., 1979), which may link to the proneness to hallucinations (Aleman et al., 2003, Moseley et al., 2016). If this were the case, it would be expected that imagined items rated high in vividness in the encoding stage of the task would be more likely to be incorrectly recalled as heard in the test stage, as demonstrated in previous studies (Johnson et al., 1988, Sugimori et al., 2014). Based on this, it would also be predicted that participants reporting high levels of hallucination-proneness would be more biased towards external misattributions (that is, have a higher misattribution bias value), and would report higher levels of vividness of imagined items. We conducted further analysis on our data, collapsed across the three experimental sessions, combining task performance data across both Experiments 1 and 2 to increase statistical power, to investigate any associations between reality monitoring performance, vividness of auditory verbal imagery, and hallucination-proneness. We therefore compared participant ratings of vividness between each of the 4 types of response (that is, 1) imagined items correctly recalled as such, 2) imagined items incorrectly recalled as heard, 3) heard items correctly recalled as such, and 4) heard items incorrectly recalled as imagined), as well as examining correlations between these ratings and hallucination-proneness. We also calculated the overall value for reality monitoring accuracy, misattribution bias, and old–new accuracy across all three sessions, and examined associations between these variables and self-reported hallucination-proneness.

### 4.1 Results

**Fig. 3:**
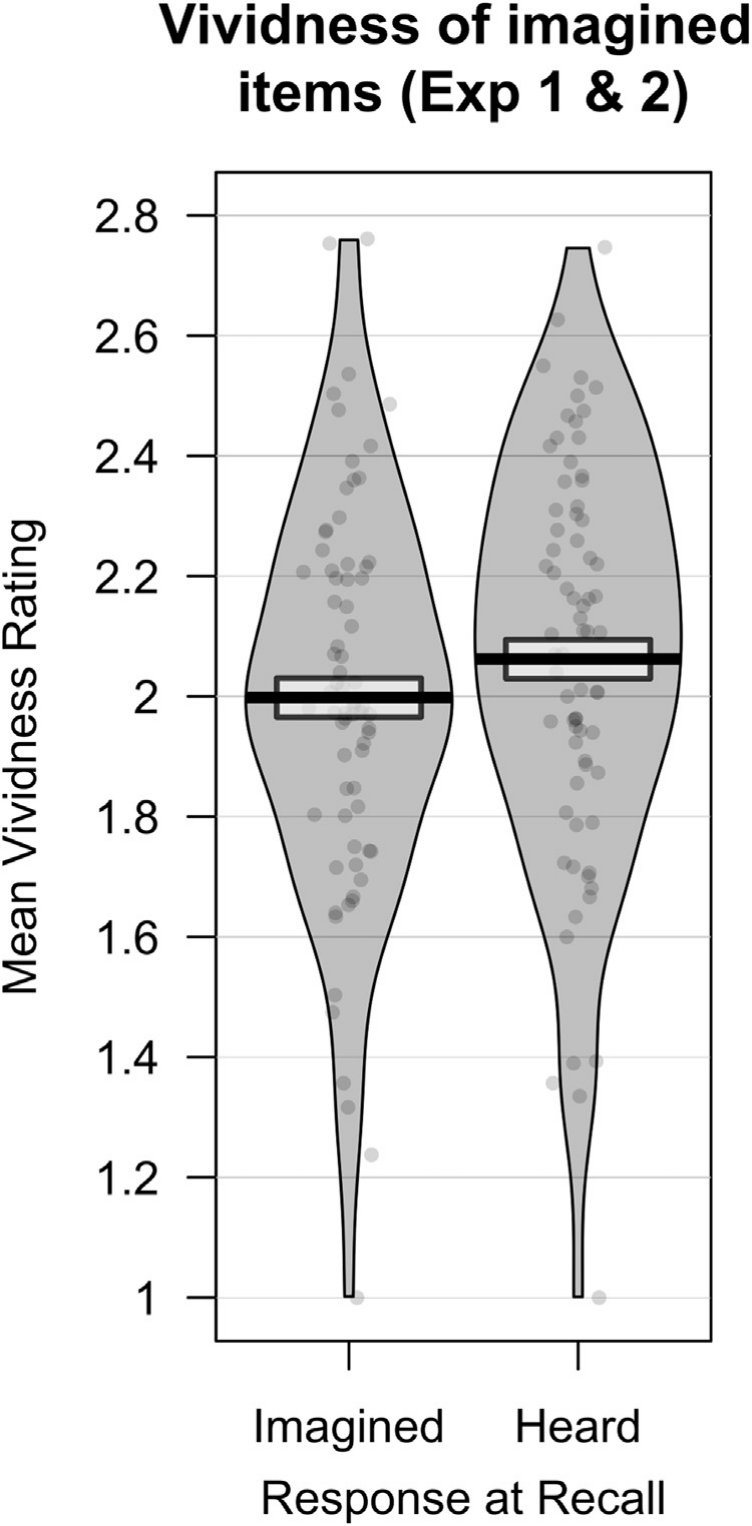
Mean vividness ratings for imagined words, categorised by participant's subsequent source judgement. Solid line = mean rating. Box = 95% confidence interval. Violin = smoothed density. Violin plots were generated in R using the ‘yarrr’ package.

A repeated measures ANOVA showed a significant main effect of response type on vividness rating (*F*(1.5, 104.9) = 193.0, *p* < .001). Post hoc *t*-tests with a corrected alpha level of 0.05 / 6 = 0.008 showed that mean vividness ratings were different between all response types. Unsurprisingly, differences in vividness between items that were heard and imagined in the encoding stage were large (Cohen's *d*'s > 1.5, all *p*'s < 0.001). Within items that were imagined in the encoding stage, items that were subsequently recalled as heard had been rated as higher in vividness than those correctly recalled as imagined (*t*(71) = 2.80, *p* = .007, *d* = 0.33). Similarly, heard items correctly recalled as such were rated as higher in vividness than those incorrectly recalled as imagined (*t*(71) = 2.81, *p* = .006, *d* = 0.33) (Fig. 3). Further analysis of the distribution of imagined items recalled as heard and those recalled as imagined using a shift function (Rousselet et al., 2017) indicated a fairly uniform shift across quantiles; that is, differences in vividness between items was similar across the distribution (although individual deciles did not cross 0 following corrections for multiple comparisons) (see Supplementary materials).

However, there was no association between any of the source memory task measures (reality monitoring accuracy, misattribution bias, old–new accuracy) and mean vividness ratings for imagined words recalled as either heard or imagined (see Table 3 for correlation coefficients). Likewise, there was no association between hallucination-proneness (as assessed by the LSHS) and any source memory task measures, or vividness ratings.

**Table 3:** Correlation matrix of source memory task performance, vividness of imagined words, and hallucination-proneness. Task performance measures represent the mean across all three sessions, whilst the vividness ratings represent the mean rating for that response type across all three sessions.

	1	2	3	4	5	6
1. Reality monitoring accuracy	–	0.120	0.713*	0.188	0.133	0.005
2. Misattribution bias		–	0.139	−0.038	−0.003	−0.153
3. Old-new accuracy			–	0.195	0.173	−0.038
4. Imagine/Imagined vividness				–	0.831*	0.143
5. Imagine/Heard vividness					–	0.081
6. Hallucination-proneness						–

* *p* < .003 (5/15).

## 5 General discussion

The two experiments reported in the present paper tested the effect of frontotemporal tDCS (as previously used in tests of the therapeutic efficacy of tDCS; e.g., Brunelin et al., 2012), compared to frontal-occipital stimulation (as used in Mammarella et al., 2017) and a sham condition, on reality monitoring. Whilst previous studies have suggested that stimulation of the STG may affect reality monitoring task performance in a healthy sample of participants (Mondino et al., 2016), and that frontotemporal stimulation may reduce AVH in patients with a diagnosis of schizophrenia (Brunelin et al., 2012), the present experiments did not provide support for this. This was the case when stimulation was applied during encoding (Experiment 1) or test (Experiment 2), using either misattribution bias or reality monitoring accuracy as the dependent variable. In both cases, the effect of stimulation failed to reach statistical significance, and further analysis using Bayes factors provided support for the null hypothesis (i.e., that there was no effect of tDCS condition on task performance). Furthermore, additional analysis using shift functions did not indicate a differential effect of tDCS on high or low task performers (using the sham condition as a baseline).

One interpretation of these findings is that the left STG and right amPFC (and associated cortical networks) may play a less important role in reality monitoring than has previously been argued. There is a large body of neuroimaging evidence suggesting that the amPFC shows increased activation during retrieval of source information using similar reality monitoring tasks, with hypothesized roles including specifically monitoring self-generated information (Mitchell and Johnson, 2009), and/or acting as a gateway between task relevant and task independent thought (Burgess et al., 2005). It could therefore be argued that this activation is epiphenomenal; that is, it is active for related processes used during reality monitoring, but is not causally necessary for encoding or source judgements. However, given that extensive neuropsychological data suggests that damage to anterior prefrontal cortex can lead to deficits in source identification (Mitchell and Johnson, 2009), this explanation seems unlikely. An alternative possibility is that tDCS applied to this cortical region does not modulate cortical excitability to a sufficient degree to affect task performance, or that other regions are capable of compensating for the mild effect of stimulation to amPFC. For example, both left and right amPFC are thought to be broadly involved in reality monitoring processes (although with potentially different roles; see Mitchell and Johnson, 2009, for an overview), and the electrodes were positioned solely over right amPFC in the present studies (though, given the medial location, it is possible that both left and right amPFC were stimulated to some extent). Stimulation of only one hemisphere may, therefore, not be sufficient to significantly affect task performance.

Although the STG is less frequently implicated in neuroimaging studies of reality monitoring, Sugimori et al. (2014) showed that activity in this region during encoding of imagined words that were subsequently recalled as heard was associated with non-clinical proneness to hallucinations; furthermore, Mondino et al. (2016) showed that left STG stimulation, applied throughout both encoding and test stages, reduced performance on a reality monitoring task (specifically leading to self-generated words to be misattributed to an external source), arguing that this effect may underlie the therapeutic effect of frontotemporal tDCS in hallucinating schizophrenia patients (Brunelin et al., 2012). Our findings therefore fail to support those of Mondino et al., although are consistent with results reported by Mammarella et al. (2017), who only showed an effect of mPFC stimulation on reality monitoring in older adults. However, it cannot be ruled out that differences in methodology may underlie the divergent findings between studies.

For example, firstly, the present study stimulated at a current strength of 1 mA with 25 cm^2^ electrodes (resulting in current density of 0.04 mA/cm^2^), whereas Mondino et al. used a stronger current (2 mA), but a larger electrode (35 cm^2^; which reduces current intensity), resulting in a current density of 0.057 mA/cm^2^. It is therefore possible that a higher current density is needed to evoke changes in task performance. The approximate density used in the present study is frequently used in tDCS studies, and has reliably evoked changes in cortical excitability in motor cortex (Horvath et al., 2015a). Doubts have been raised, however, regarding the capability of tDCS to evoke changes in cognitive task performance, with one meta-analysis finding no convincing evidence of any change in cognitive task performance following tDCS (Horvath et al., 2015b; though see Price et al., 2015). Further research, including the publication of ‘null’ results from tDCS studies, is needed to provide clarity on this issue.

Secondly, the two experiments presented here used stimulation separately during the encoding and test phases, respectively. In contrast, the experiment reported by Mondino et al. (2016) stimulated throughout both stages of the task. It is possible that the combined effect of stimulation in both parts of the task increased the effect on task performance; this is a possibility that should be tested in future studies. However, Mammarella et al. (2017) stimulated during only the encoding stage of a reality monitoring task, implying that stimulation throughout the task may not be necessary.

Thirdly, Mondino et al. (2016) did not use a frontotemporal montage, instead opting to stimulate prefrontal and temporal regions separately. The montage in the present study was chosen based on the use of tDCS in studies of its therapeutic efficacy, which have stimulated frontal and temporal regions concurrently. Finally, a difference in the method used to locate STG/TPJ may account for our divergent findings: while Mondino et al. placed the anodal electrode over the midpoint between T3 and P3 (using the EEG 10–20 system), the present study placed the electrode over location CP5 (using the 10–10 system). However, the difference between these electrode locations is likely to be very small, especially given the relatively large electrodes used with tDCS (25 cm^2^). We would, therefore, argue that this methodological difference is unlikely to account for the difference in findings between the two studies.

One argument might be that tDCS shows differential effects depending on task proficiency, or the state of the brain at stimulation onset (Benwell et al., 2015). One possibility could therefore be that participants who show lower performance in reality monitoring tasks would be more affected by stimulation (which may explain the purported efficacy of tDCS to have therapeutic effects in patients with AVH – a population that show deficits in reality monitoring; Brookwell et al., 2013). However, our analysis using shift functions suggested no difference in the distribution of data across stimulation conditions, compared to the sham condition, seemingly ruling this explanation out. This is an important area for future tDCS research to investigate further. Our findings therefore contrast with those of Mondino et al.; clearly, further research is needed to clarify whether tDCS applied to the STG can affect reality monitoring.

Further analysis of the task data, across both studies combined, indicated that self-reported vividness of imagined words, was associated with their subsequent recognition as heard or imagined, at test. Specifically, imagined words that were subsequently recalled as heard were reported as higher in vividness in the encoding stage of the task. This replicates previous research (Johnson et al., 1988, Sugimori et al., 2014), and is consistent with theorising within the source monitoring framework suggesting that a crucial aspect of reality monitoring is using qualitative aspects of remembered information (in this case, perceived vividness) to inform source judgements. It has previously been hypothesized that excessively vivid auditory verbal mental imagery may be associated with AVH, both in clinical populations, but also in non-clinical participants prone to hallucinations (Aleman et al., 2003). The present study found no association between vividness of imagined words and hallucination-proneness; however, there was also no significant association between any aspect of task performance and hallucination-proneness. Although some studies have shown associations between reality monitoring and non-clinical hallucination-proneness (Laroi et al., 2004), a recent study failed to find this association (Garrison et al., 2017). Deficits in reality monitoring have been shown in multiple studies comparing hallucinating and non-hallucinating schizophrenia patients, however (see Brookwell et al., 2013, for a meta-analysis), raising the possibility that reality monitoring deficits are only associated with hallucinations in clinical populations.

An important finding from the experiments was that participants were relatively successful at guessing the sham condition (44/72 over both experiments, compared to the 24/72 that would be expected by chance). Participants correctly chose the sham condition significantly above chance in Experiment 1, but not in Experiment 2, although the difference between accuracy rates in the two experiments was small (24/36 in Experiment 1, 20/36 in Experiment 2). It should be noted, though, that even participants who correctly guessed the sham condition were not highly confident in their decision. It seems unlikely that the ineffectiveness of the sham condition can explain the null results reported, as there were two active stimulation conditions, with no observable difference between task performance when different regions were stimulated. However, further research should be conducted into the efficacy of sham stimulation to act as a realistic control condition (O’Connell et al., 2012), in particular regarding what evidence participants may use to judge one session as sham (e.g., physical sensation on scalp, or experimenter behaviour in a single-blind study). Sham stimulation in tDCS experiments is often assumed to be effective due to the relatively mild sensation underneath the electrodes, and the lack of noise emitted by the equipment during stimulation (unlike, for example, TMS), but further research should explore the conditions under which this assumption does and does not hold.

Due to the constraints of running a tDCS study across multiple sessions, the reality monitoring task differed in a number of ways from previous research. For example, many reality monitoring studies use memory paradigms in which the participant is unaware they will be tested for recall of source information. However, it was not possible to blind the participant to this aspect of the task, given that they completed the task on three separate occasions (in the three different stimulation conditions). Participants in the present study were therefore instructed that they would be asked to recall the source of the verbal stimuli in their initial task instructions. It is possible that this could have affected the strategy that participants used to complete the task. Although Mondino et al. (2016) do not explicitly state that participants were informed of the test stage beforehand, we assume that this was the case, since a similar within-subjects design was used in their study. Similarly, we used a source memory paradigm in which participants were either instructed to listen to or imagine verbal stimuli, as opposed to some previous studies which have required the participant to speak the word aloud. It could be argued that participants may not have always followed the instruction to imagine specific words, therefore affecting task performance. That said, our findings showing that self-reported imagery vividness was associated with subsequent source judgement imply that participants were following instructions. Previous source memory studies (e.g., Mondino et al., 2016; Sugimori et al., 2014) have also successfully used ‘hear-imagine’ paradigms. We therefore contend that our findings are still directly comparable to previous research (despite the methodological details outlined above).

A further limitation of the present study is that there was no ‘positive’ control condition; that is, a condition in which the tDCS protocol was shown to evoke changes in another behavioural task. Inclusion of such a condition would have allowed stronger conclusions regarding the involvement (or lack of involvement) of specific cortical regions in the reality monitoring task. However, we argue that such a condition is not practically feasible, because there is no behavioural paradigm that is strongly and reliably affected by frontotemporal stimulation (see above, Horvath et al., 2015b). It is also possible that the anodal montage over occipital areas might have induced a small amount of current flow in the STG, although models of current distribution in tDCS show that current density is the strongest in the areas directly under electrodes, and decreases with distance from areas where stimulation was applied (Miranda et al., 2006, Wagner et al., 2007). As noted, presentation of heard and imagined items was not fully randomised, in order to ensure that different encoding conditions were balanced across time during stimulation, which may have affected the encoding strategies used by participants. Finally, our samples were biased towards the female gender. Some studies have shown differential effects of tDCS between genders (Chaieb et al., 2008), giving reason for concern that our findings might not generalise to males.

As such, following the results presented here, our conclusion is that either tDCS does not reliably affect cortical excitability in frontal or temporal regions (when using the protocol presented in this paper), or that these regions play a less crucial role in reality monitoring than has been previously argued. Further research is needed to discriminate between these, for example combining tDCS with fMRI to provide data on the neural effects of frontotemporal stimulation.

To summarise, the present study showed no effect of frontotemporal or frontal-occipital tDCS, in contrast to previous findings. Combining data across two studies, further analysis showed externally misattributed imagined items were previously rated as higher in vividness compared to other items, supporting previous behavioural findings. However, no task performance measures were associated with self-reported hallucination-proneness, suggesting that reality monitoring deficits may be associated with hallucinations in clinical populations, but not with proneness to hallucinations in the general population. Further research should be conducted into the efficacy of tDCS to modulate task performance in cognitive tasks such as reality monitoring, in both clinical and non-clinical populations, and also into the neural basis of proneness to hallucinations in the general population, as compared to clinical populations.
