# Supplementary material for: Investigating the roles of medial prefrontal and superior temporal cortex in source monitoring
Source: Neuropsychologia. 2018 Nov;120:113–23. doi: 10.1016/j.neuropsychologia.2018.10.001 (PMC6227377; doi:10.1016/j.neuropsychologia.2018.10.001)
Supplement: Supplementary file 1 — Supplementary material [file mmc1.docx]

**Supplementary materials**

The supplementary materials below report the results of the shift functions applied in the main text in more detail. Shift functions are described in more detail in the main text, and in particular in Rousselet, Pernet, & Wilcox (2017). Briefly, scores are compared at each decile, as a function of the decile of one group, enabling a simple method of investigating differences in distributions of different conditions. In the below plots, a relatively flat line across the deciles represents a uniform difference (or lack of difference) between conditions, whereas a slanted line would represent that the difference between groups may be highest at one point of the distribution. For all plots, error bars represent 95% confidence intervals corrected for multiple comparisons. All code and shift function data are available at: osf.io/7nr4v


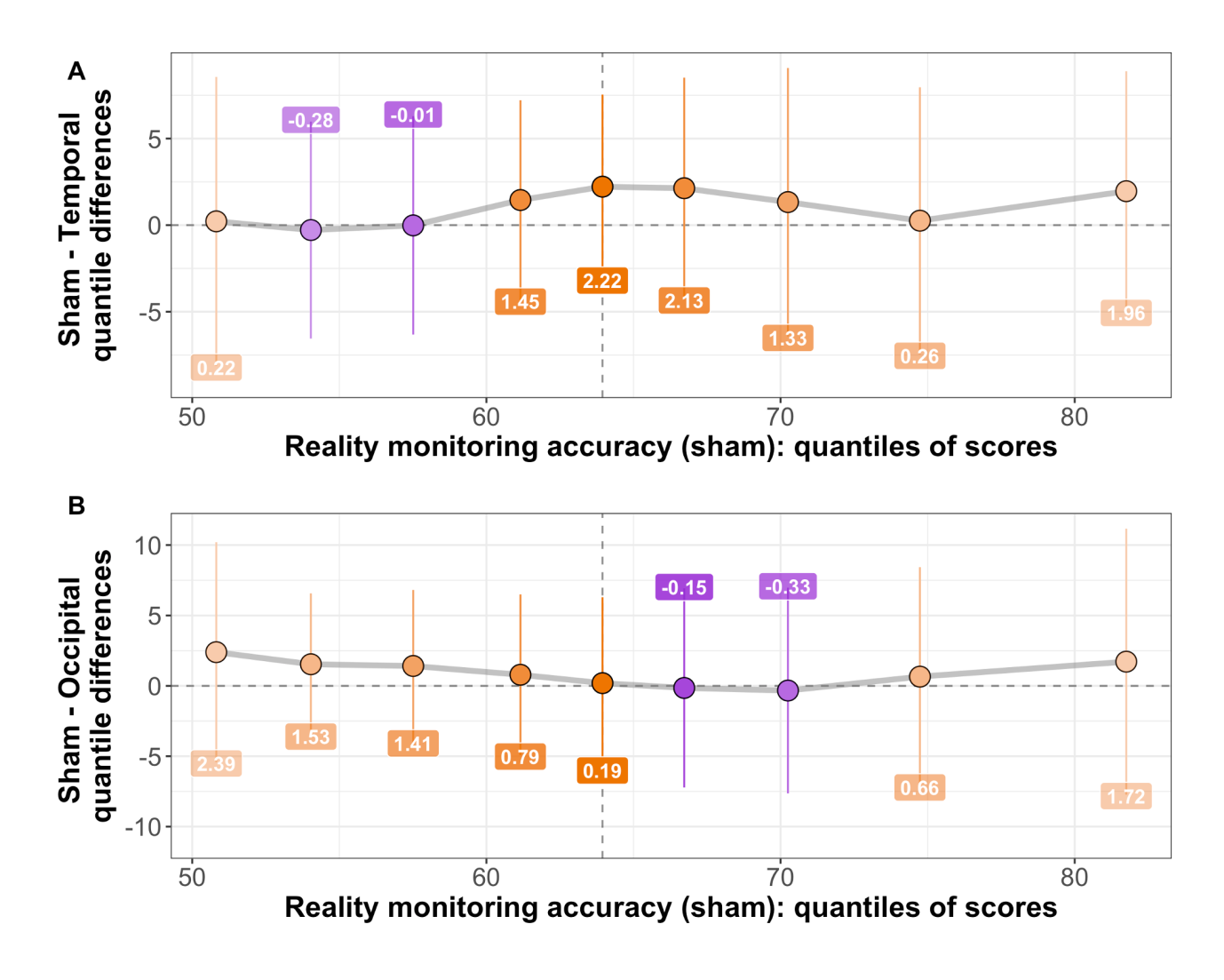


S1: Shift function plots for reality monitoring accuracy for (A) frontotemporal and (B) frontal-occipital conditions, using sham as a baseline, in Experiment 1.


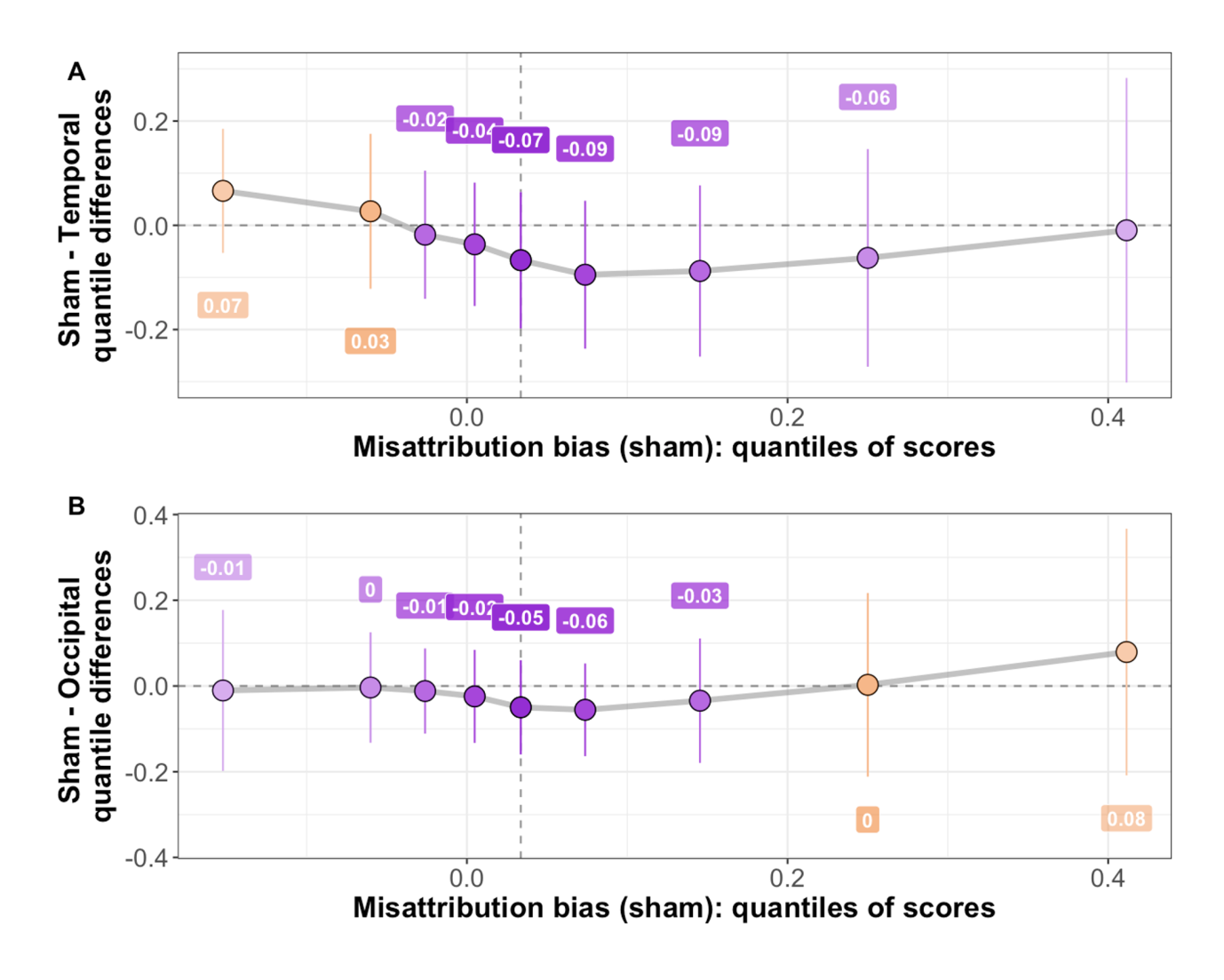
S2: Shift function plots for misattribution bias for (A) frontotemporal and (B) frontal-occipital conditions, using sham as a baseline, in Experiment 1.


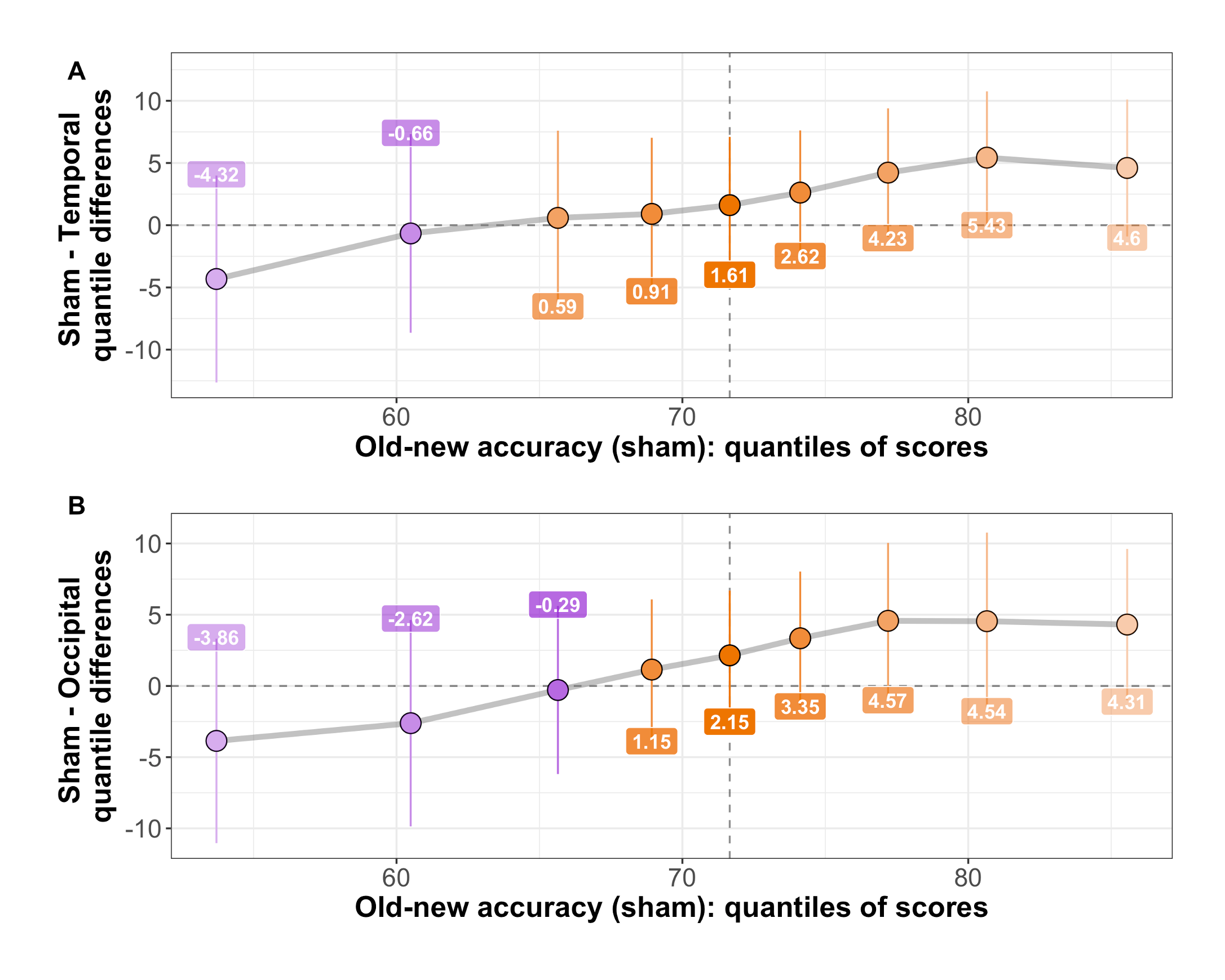


S3: Shift function plots for old-new accuracy for (A) frontotemporal and (B) frontal-occipital conditions, using sham as a baseline, in Experiment 1.


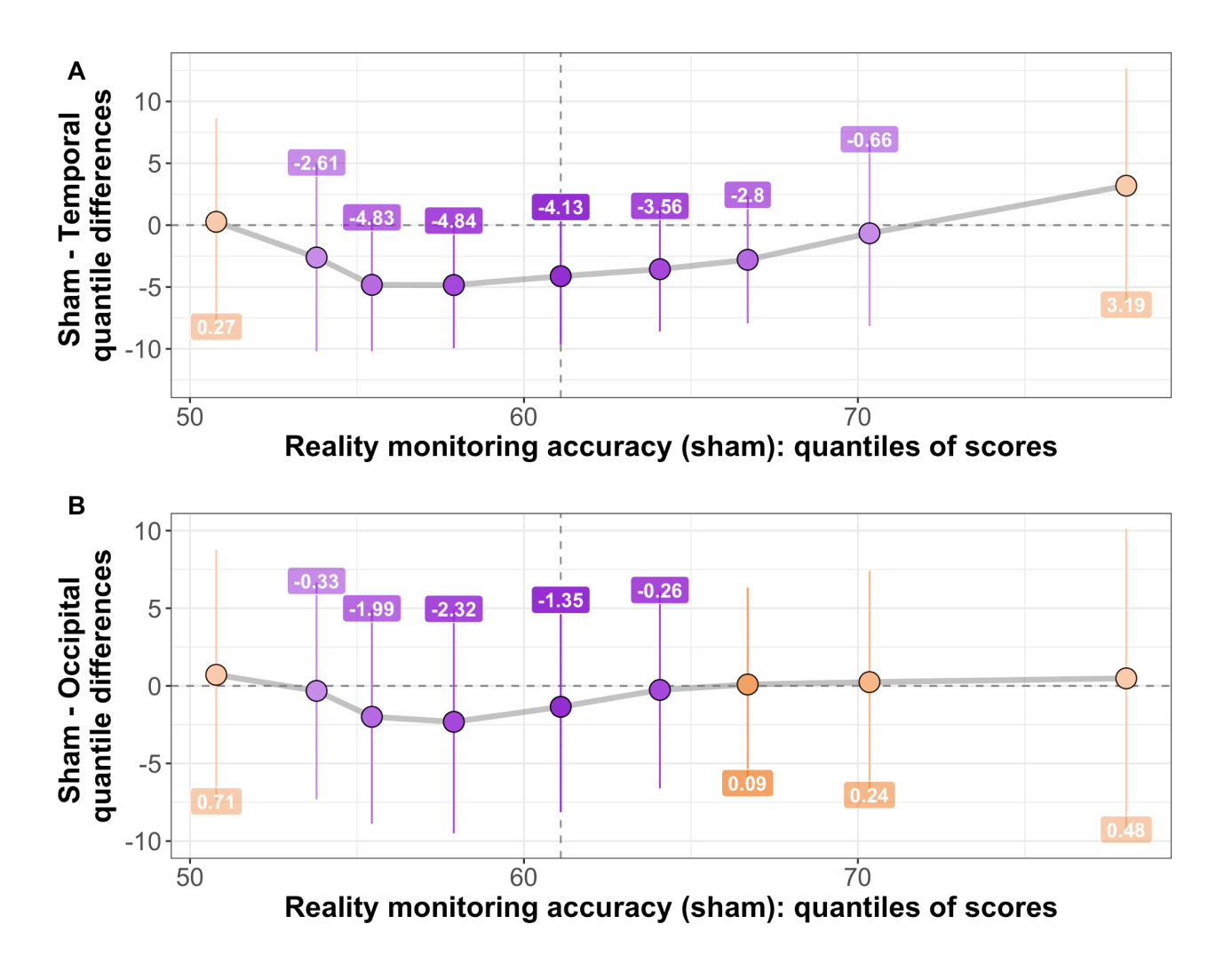


S4: Shift function plots for reality monitoring accuracy for (A) frontotemporal and (B) frontal-occipital conditions, using sham as a baseline, in Experiment 2.


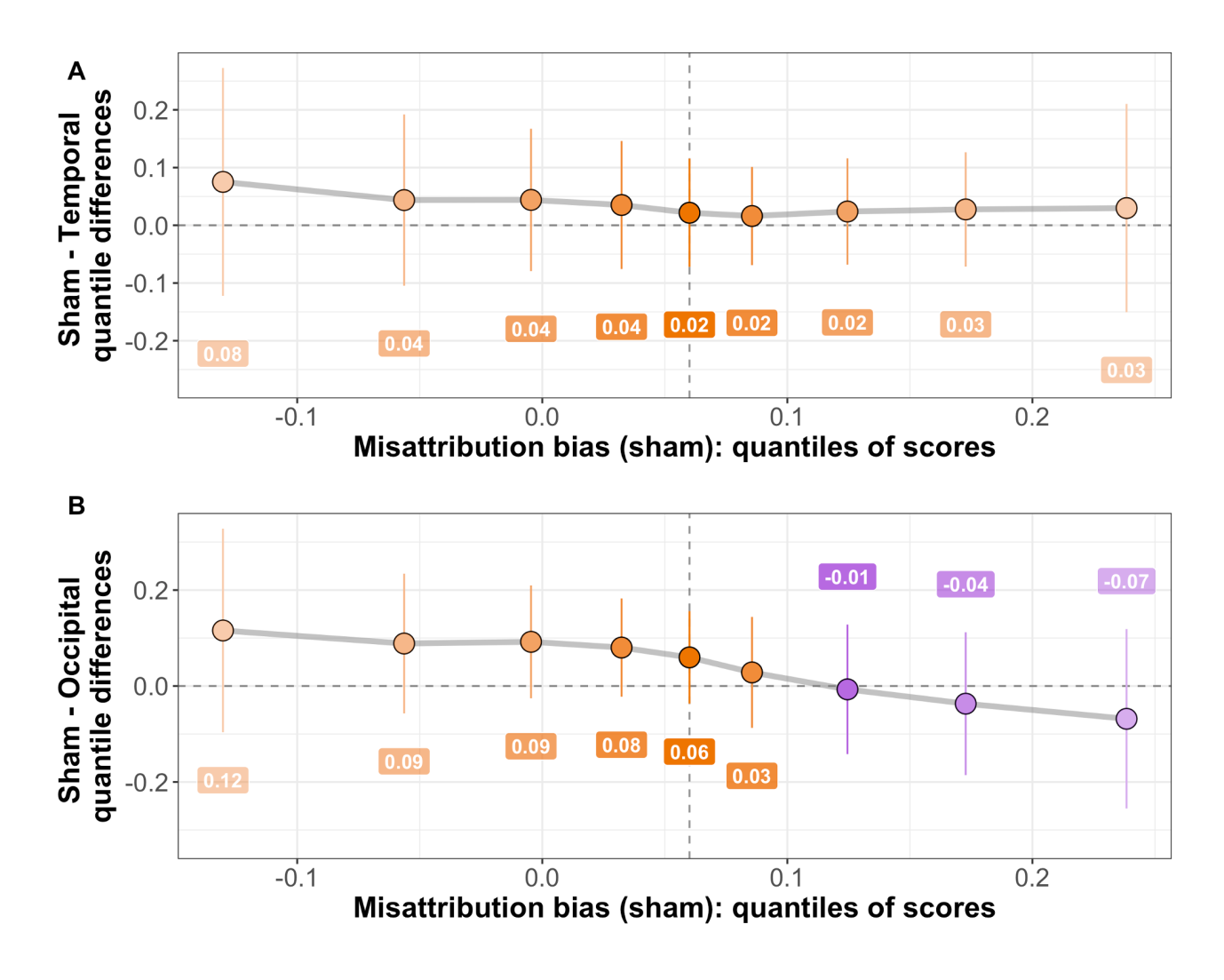


S5: Shift function plots for misattribution bias for (A) frontotemporal and (B) frontal-occipital conditions, using sham as a baseline, in Experiment 2.


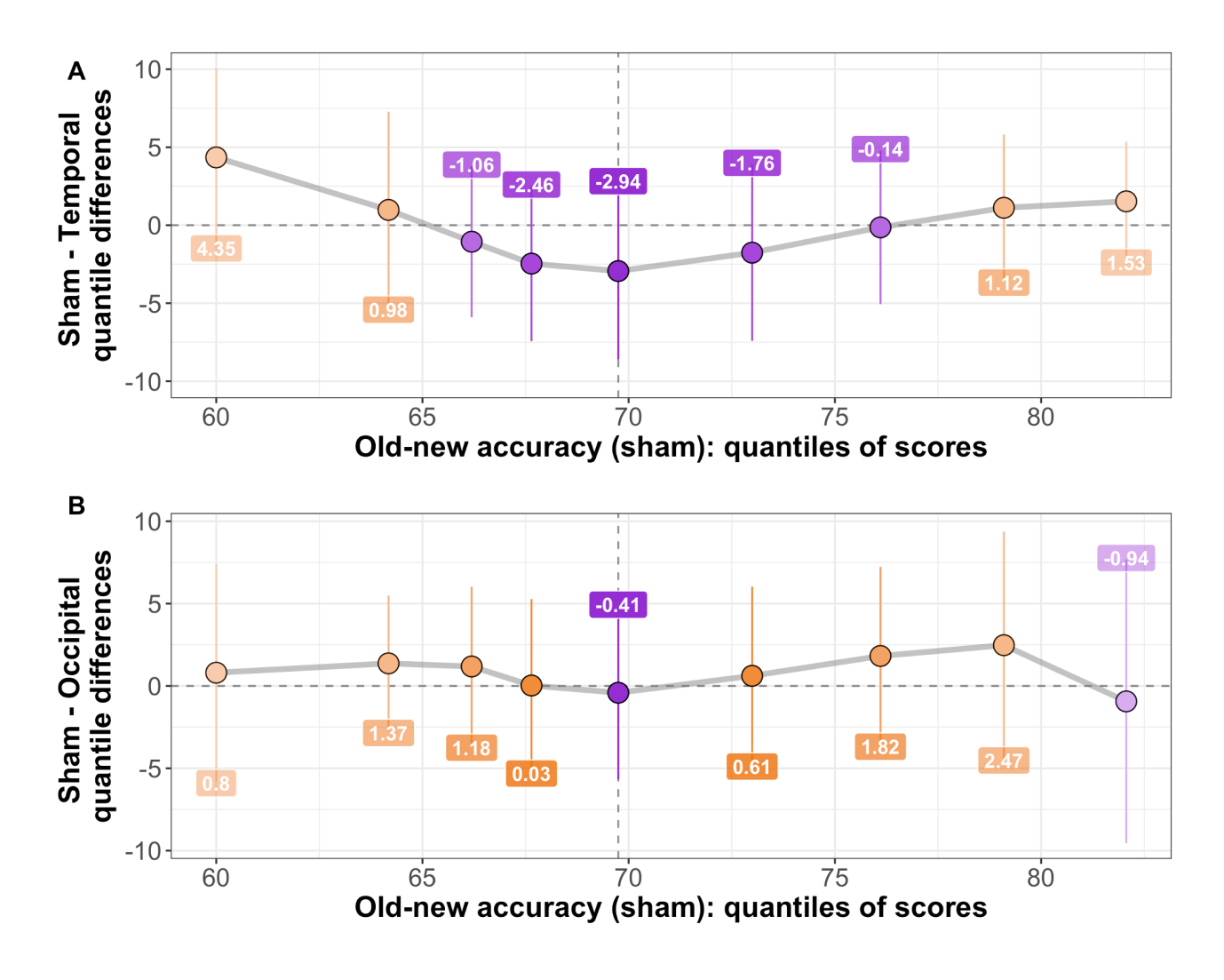


S6: Shift function plots for old-new accuracy for (A) frontotemporal and (B) frontal-occipital conditions, using sham as a baseline, in Experiment 2.


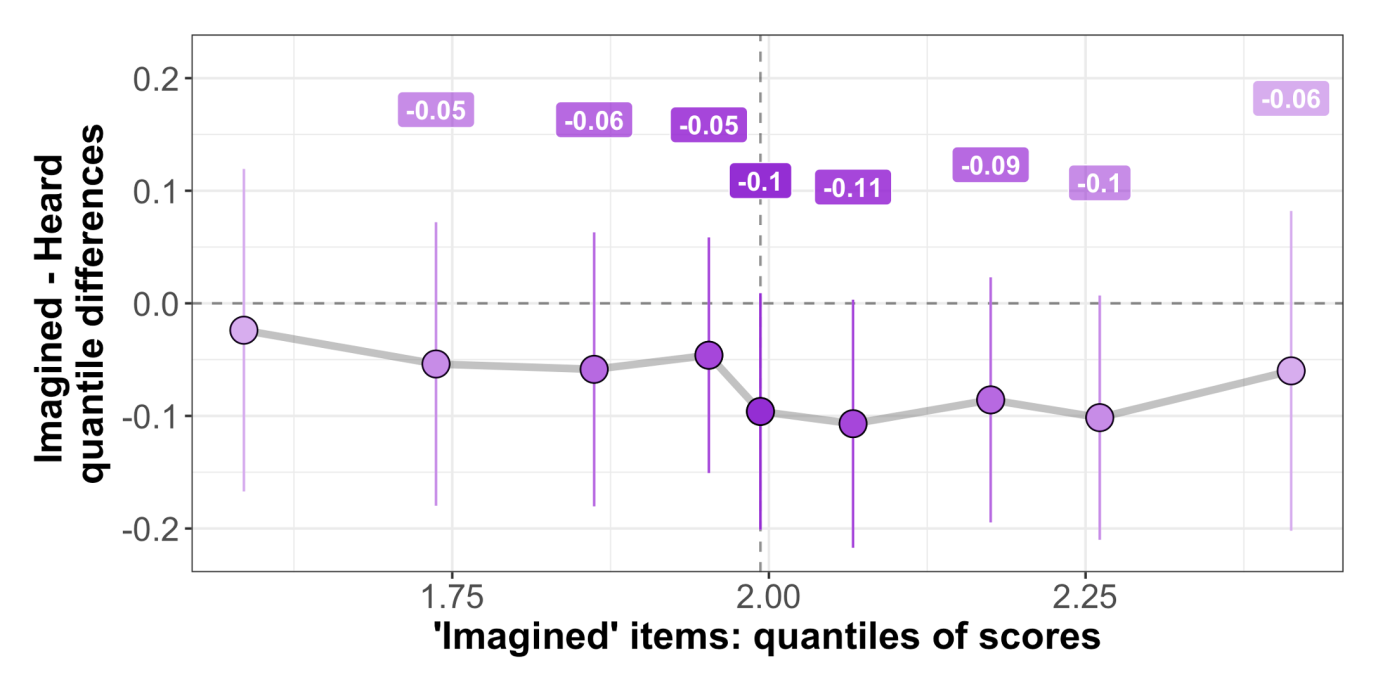


S7: Shift function plots for word vividness ratings, specifically for words that participants were instructed to ‘imagine’ in the encoding stage. Here, words that were incorrectly recalled as heard are compared to those recalled as imagined, with data collapsed across both experiments.
